# Haematopoietic Cells Deficient for Calreticulin or Expressing Its Oncogenic Variants Maintain Functional Antigen Presentation

**DOI:** 10.1111/jcmm.71399

**Published:** 2026-10-11

**Authors:** Maria Höhrhan, Laura Antonio‐Herrera, Henrique G. Colaço, Anja Wagner, Markus Unterwurzacher, Cecilia Varga, Klaus Kratochwill, Andreas Bergthaler, Robert Kralovics

**Affiliations:** ^1^ Department of Laboratory Medicine Medical University of Vienna Vienna Austria; ^2^ Research Center for Molecular Medicine of the Austrian Academy of Sciences (CeMM) Vienna Austria; ^3^ Institute for Hygiene and Applied Immunology Medical University of Vienna Vienna Austria; ^4^ Core Facility Proteomics Medical University of Vienna Vienna Austria

**Keywords:** antigen presentation, calreticulin, MHC class I, myeloproliferative neoplasms, peptide loading complex

## Abstract

Intact antigen presentation in cancer cells is a key prerequisite for endogenous anticancer T cell responses and a starting point for immunotherapy. Calreticulin was postulated as a chaperone and integral part of the peptide‐loading complex of MHC‐I. Oncogenic mutations of calreticulin allow surface expression in complex with the thrombopoietin receptor, leading to ligand‐independent activation, cell proliferation, and myeloproliferative neoplasms. We hypothesized that mutated calreticulin could also corrupt the function of MHC‐I, as previous reports revealed decreased surface expression, peptide‐loading defects and retention of MHC‐I in the Golgi apparatus. We employed reporter cell models that utilized the T cell response towards target murine and human primary cells and cell lines carrying calreticulin mutations as a functional readout. We also harnessed T cell responses upon a viral infection In Vivo. We found no evidence for immunosuppression in mutant calreticulin‐expressing or calreticulin‐deficient cells. Our study implies an overestimation of the significance of calreticulin for MHC‐I functionality and provides an impulse for reevaluation. Furthermore, we show that calreticulin deficiency affects only a few proteins, including myeloperoxidase and other proteins involved in antibacterial immune mechanisms. In conclusion, mutant calreticulins do not interfere with antigen presentation in MPN, making MHC‐I‐dependent immunotherapies a viable venue for treatment.

## 1 Introduction

Calreticulin was first characterized as an endoplasmic reticulum (ER)‐residing protein involved in calcium storage [1, 2]. More importantly, calreticulin became known as a lectin‐like chaperone and part of the peptide loading complex (PLC) of major histocompatibility complex class I (MHC‐I) [3, 4, 5, 6, 7].

Somatic mutations in the calreticulin (CALR) gene lead to expression of oncogenic calreticulin variants (oncoCALR) and thus contribute to the pathogenesis of certain myeloproliferative neoplasms (MPN) [8, 9]. OncoCALR loses its ER‐retention signal KDEL, which allows it to be secreted or surface‐expressed in complex with the thrombopoietin receptor (MPL). The oncoCALR/MPL interaction triggers ligand‐independent activation of MPL and leads to clonal expansion of mutated haematopoietic stem cells and their progeny [10, 11, 12, 13].

The MHC‐I surface expression level decreases in oncoCALR‐expressing cells, and they exhibit multiple defects of peptide loading [14, 15]. The dependency of certain MHC‐I‐presented peptide levels on calreticulin was reported [16, 17]. Loading of subpar peptides and accumulation of MHC‐I in the Golgi apparatus were also revealed after loss of the KDEL sequence of calreticulin [18]. Calreticulin‐deficient cells were found to be unable to present certain peptides and expressed an altered MHC‐I repertoire on the cell surface [16, 17, 19, 20].

Immunopeptidomics revealed a lack of peptides derived from oncoCALR despite predicted presentability [21, 22].

Thus, we hypothesized that oncoCALR could not only corrupt the function of MPL but also that of MHC‐I, making it a unique oncogene that simultaneously induces oncogenesis and immune evasion.

We aimed to dissect the interaction of oncoCALR and MHC‐I and assess whether and how the reported defects influence the functionality of MHC‐I in the immune response in vitro and In Vivo. Therefore, we profiled MHC‐I function through a combination of in vitro models (e.g., co‐culture assays of reporter cells with various target cells derived from murine primary cells and cell lines carrying calreticulin mutations, as well as MPN patient cells) and an In Vivo viral infection model.

Surprisingly, we found no evidence for MHC‐I dysfunction in oncoCALR‐expressing or calreticulin‐deficient cells and mice.

To pinpoint the actual role of calreticulin in the proteome on a molecular level, we performed tandem mass tag mass spectrometry (TMT‐MS) on calreticulin‐deficient and oncoCALR‐positive primary murine neutrophils and T cells, thereby looking for proteins missing in the mutated cells. We revealed that calreticulin is an indispensable chaperone for a subset of proteins, but not MHC‐I.

We thus conclude that calreticulin—despite textbook knowledge assigning it a vital role in the PLC—is not an essential contributor to MHC‐I functionality in the examined alleles [23, 24].

## 2 Methods

### 2.1 Cell Lines

Founding cells of the pro‐B cell line Ba/F3 were isolated from the bone marrow of a female C3H mouse. Our lab previously engineered those cells to express various oncogenic calreticulin variants and a knockout of calreticulin [25]. Cells were cultured in RPMI, 10% FBS, 2% WEHI‐3B supernatant, 100 U/mL penicillin, and 100 μg/mL streptomycin (P/S).

The Jurkat 1E6 cell line is a cancer cell line that was obtained from a male juvenile patient suffering from T acute lymphoblastic leukaemia. Jurkat cells were maintained in RPMI, 10% FBS, 100 U/mL penicillin, and 100 μg/mL streptomycin (P/S).

UT‐7 cells were first obtained from an elderly male patient with acute megakaryoblastic leukaemia. Cells were cultured in IMDM, 10% FBS, 5% TPO supernatant, 100 U/mL penicillin, and 100 μg/mL streptomycin (P/S).

### 2.2 Genetic Engineering

Retro‐ and lentiviral plasmids were based on pMSCV and pCDH vector backbones, respectively. Primers were ordered from Sigma–Aldrich; fragments were amplified by PrimeSTAR Max DNA Polymerase (Takara), assembled by NEBuilder HiFi DNA Assembly Master Mix (NEB), and constructs were validated by Sanger sequencing.

Virus was produced in HEK293T‐derived cell lines maintained in DMEM, 10% FBS, 1% GlutaMax after transfection with calcium phosphate [26]. Retrovirus was produced by the PlatE cell line (Cell Biolabs), while lentivirus was produced by LentiX cells (Takara) using helper plasmids that were gifts from Didier Trono (Addgene #12251, #12253, and #12259). Equal amounts (μg) of each helper plasmid were used, with the vector plasmid used at twice the amount of each helper plasmid. A glycerol shock and medium change were performed the day after transfection, and virus supernatant was harvested 24 h and 48 h later.

For transduction, virus supernatant was diluted with RPMI containing 10% FBS at a 1:1 ratio, and 8 μg/mL polybrene was added. 10^6^ cells/well in a 6‐well plate were resuspended in virus mix and centrifuged twice (670 × g at 32°C 90 min) with fresh virus mix added the second time. Cells were incubated with the virus mix supplemented with respective cytokines overnight, and the medium was changed the next day.

### 2.3 Mouse Strains

The Austrian Federal Ministry of Education, Science and Research approved all described experimental procedures in animal experimentation licences 2020–0.448.862 and 2020–0.406.011. Mice were bred and housed in specific‐pathogen‐free (SPF) conditions in our mouse facility and received water and chow ad libitum.

C57BL/6N mice expressing CALRdel52 in their haematopoietic system under a vavCre promoter were previously generated in our lab and bred with homo‐ and heterozygously expressed transgene [27]. We bred vavCre‐negative individuals carrying the CALRdel52 transgene with AlbCre‐positive mice to obtain transgene expression in the liver [28].

C57BL/6N mice carrying a conditional calreticulin knockout were bred with vavCre mice to obtain mice deficient in calreticulin in the haematopoietic system [29, 30].

OT‐I mice were bred as homozygotes [31].

### 2.4 T Cell Isolation and Expansion

OT‐I mice were euthanized by CO2 and spleens were harvested. Single‐cell solutions of splenocytes were prepared and frozen. For experiments, OT‐I splenocytes were expanded as described [32]. In brief, splenocytes were thawed and immediately applied to an activating antibody‐coated surface (0.5 μg/mL anti‐mCD3 and 5 μg/mL anti‐mCD28 antibody, both from Biolegend), cultured in RPMI, 10% FBS, 1X insulin transferrin selenium supplement, 50 μM beta‐mercaptoethanol and P/S. 30 U/mL rmIL‐2 (Biolegend) was added starting from day 2. On day 3, the medium was changed and cells were transferred into a fresh vial. Half of the medium was changed and 30 U/mL rmIL‐2 was replenished every day. T cells were expanded for 9–10 days before use. This method yields a mixed cell population comprising approximately half‐and‐half CD4+ and CD8+ T cells; cell numbers for subsequent experiments were calculated accordingly.

### 2.5 Isolation and Culture of Murine Haematopoietic Progenitor Cells

Mice were euthanized by CO2, and humerus, tibia, femur, and hip were extracted. Bone marrow was obtained by crushing the bones with a mortar and pestle in sterile conditions. Haematopoietic progenitor cells were isolated using the MojoSort Mouse Hematopoietic Progenitor Cell Isolation Kit (Biolegend) and frozen thereafter. For experiments, haematopoietic progenitor cells were maintained in RPMI, 10% FBS, 20 ng/mL mFlt‐3 L, 10 ng/mL rmIL‐3, 10 ng/mL rmIL‐6, 20 ng/mL mSCF, and P/S (all cytokines obtained from Immunotools) [33].

### 2.6 Cell Sorting

Transduced Ba/F3 cells were isolated by magnetic‐activated cell sorting (MACS) employing the Dynabeads FlowComp Flexi Kit (Invitrogen) and biotinylated anti‐H‐2Kb antibody (Biolegend). Transduced primary haematopoietic stem cells and the CAR Jurkat cell line were FACS sorted.

### 2.7 Flow Cytometry

Murine samples from blood, spleen, bone marrow, or co‐culture experiments were prepared for flow cytometry using Zombie Violet fixable viability dye (Biolegend) and anti‐mouse antibodies B2m‐PE, Viability‐APC‐Cy7, CD11b‐FITC, GP33‐PE, CD3‐PE‐Cy7, CD8‐APC, CD44‐BV605, CD62L‐FITC, CD4‐BV605, CD8‐PECy7, CD3‐PECy5.5, Ifng‐FITC, Gzmb‐APC, TNF‐PE (Biolegend), H‐2Kb‐APC and CD8a‐APC (eBioscience).

Human cells were stained using Zombie Green fixable viability dye (Biolegend), and antibodies against CD69‐APC and HLA‐PacificBlue (Biolegend), as well as anti‐G4S‐linker‐AlexaFluor647 (Cell Signaling Technology). Intracellular staining was performed using Intracellular Staining Perm Wash Buffer (Biolegend). Samples were analysed employing a BD LSRFortessa equipped with an HTS autosampler and evaluated by BD FlowJo v10 software.

### 2.8 Co‐Culture

For co‐culture experiments, effector and target cells were co‐cultured at an effector‐to‐target (E:T) ratio of 1:1. During co‐culture, cells were maintained in the medium of the target cells. Ba/F3 cells were co‐cultured for 24 h, CD34+ cells for 48 h and haematopoietic stem cells for 72 h, after empirical determination of incubation times.

### 2.9 Western Blot

Murine PBMCs were lysed in RIPA buffer (Thermo Fisher Scientific); the lysate was loaded onto an SDS‐PAGE gel and proteins were transferred to a nitrocellulose membrane (Cytiva Amersham). Calreticulin was detected using chicken anti‐calreticulin polyclonal serum and anti‐IgY‐HRP antibody (Abcam).

### 2.10 In Vivo Viral Clearance

Mice were divided into sex‐ and age‐matched groups of five, thereby generating mixed‐sex groups. Acute lymphocytic choriomeningitis virus (LCMV) strain Armstrong was grown on BHK‐21 cells and titrated by focus‐forming assay [34]. Mice were infected with LCMV by tail vein injection of 2 × 10^6^ focus‐forming units (ffu) of virus supernatant. During infection, the health status of mice was monitored by assessment of body weight on day 0, 3 and 8. Eight days post‐infection, T cell responses in the blood and spleen were assessed by flow cytometry. For intracellular staining of cytokines in CD8+ T cells, we restimulated them by incubation with 1 μg/mL of the LCMV gp33–41 peptide at 37°C for 4 h in the presence of a protein transport inhibitor cocktail (Thermo Fisher Scientific), containing brefeldin A and monensin.

### 2.11 Isolation and in vitro Culture of CD34+ Cells

PBMC samples from MPN patients and healthy control donors were collected with informed consent during the generation of our biobank under the approval number #553/2008 of the ethics committee of the Medical University of Vienna. Patients were included in this study after detection of oncoCALR and the HLA‐A*02:01 allele by RNA sequencing [21]. Healthy control donors were identified after isolation of genomic DNA from saliva using the DNeasy Blood and Tissue Kit (Qiagen) followed by genotyping for the HLA‐A*02:01 allele by PCR [35].

We isolated CD34+ haematopoietic cells from frozen PBMCs using the EasySep Human CD34 Positive Selection Kit II and subsequently cultured them in StemSpan SFEM II medium supplemented with StemSpan CD34+ Expansion Supplement (STEMCELL Technologies), P/S, and normocin (InvivoGen). Cells were expanded for 7 days before use.

### 2.12 Gene Expression Analysis

Expression levels of WT1 and PD‐L1 in healthy and patient CD34+ cells and the UT‐7 cell line were assessed by quantitative PCR. Therefore, RNA was isolated using the RNeasy Mini Kit (Qiagen) and reverse‐transcribed to cDNA using the LunaScript RT SuperMix Kit (NEB). qPCR was performed using the Luna Universal qPCR Master Mix (NEB) and WT1‐specific primers as published [36]. Origene provided primer sequences for PD‐L1 and the housekeeping gene RPS18.

### 2.13 Analysis of the Proteome

C57BL/6N mice carrying CALRdel52 in the homozygous state, a *Calr* knockout or *Calr* WT were euthanized by CO2. Neutrophil granulocytes were isolated from the bone marrow and cytotoxic T cells from the spleen using the MojoSort Mouse Neutrophil Isolation Kit and the MojoSort Mouse CD8+ T Cell Isolation Kit (Biolegend).

Whole cell lysates were prepared using lysis buffer (30 mM Tris, 7 M urea, 2 M thiourea, 4% CHAPS, 1 mM EDTA, one tablet of Complete Protease Inhibitor (Roche), and one tablet of Phosphatase Inhibitor (PhosSTOP, Roche) per 100 mL pH 8.5). Protein concentration was measured using the Pierce 660 nm assay according to the manual.

Protein digestion of whole cell lysates was performed using single‐pot, solid‐phase enhanced sample preparation (SP3). Briefly, the reduced and alkylated proteins were bound to SP3 beads (10:1 beads:protein ratio, GE Healthcare) and subjected to on‐bead digestion with trypsin/LysC (1:25 protease: protein ratio, Promega).

Resulting peptides were desalted using Pierce Peptide Desalting Columns (Thermo Fisher Scientific), dried and reconstituted in TEAB (Fluka). Peptide concentration was determined using the Colorimetric Peptide Assay (Thermo Fisher Scientific).

TMTpro labelling (Thermo Fisher Scientific) was performed according to the manufacturer's protocol. Labelling efficiency was determined by analysis of a small aliquot of each sample by LC–MS and ProteomeDiscoverer.

Labelled samples were concentrated and desalted with Pierce Peptide Desalting Columns (Thermo Fisher Scientific) and eluates were dried and reconstituted in ammonium formate buffer.

Two‐dimensional liquid chromatography was performed by reverse‐phase chromatography at high and low pH, as previously described [37]. In the first dimension, peptides were separated on a Gemini‐NX C18 (Phenomenex) in ammonium formate buffer and eluted with a gradient using an Ultimate 3000 RSLC micro system (Thermo Fisher Scientific) equipped with a fraction collector. Thirty‐six fractions were collected in a time‐based manner (every 30 s from min 11.5–57). Organic solvent was removed and samples were reconstituted in 0.1% TFA.

Fractions were analysed on an Ultimate 3000 RSLC nano coupled directly to an Exploris 480 with FAIMSpro (all Thermo Fisher Scientific). Samples were injected onto a reversed‐phase C18 column (50 cm × 75 μm i.d., packed in‐house) and eluted with a gradient of 4%–38% mobile phase B over 94 min by applying a flow rate of 230 nL/min.

MS scans were performed in the range from m/z 375–1650 at a resolution of 120,000 (at m/z = 200). MS/MS scans were performed at a resolution of 30,000 with turboTMT mode; normalized collision energy of 33%; isolation width of 0.7 m/z and dynamic exclusion of 90 s. FAIMS was operated in standard resolution mode with a static carrier gas flow of 4.6 L/min, two FAIMS voltages (–40 V and –60 V) and a cycle time of 1.5 s per voltage.

The acquired raw MS data files were processed and analysed using ProteomeDiscoverer (v2.4.0.305, Thermo Fisher). SequestHT was used as the search engine and the following parameters were chosen: database: *Mus musculus* (SwissProt, downloaded on 2024‐01‐26); enzyme: trypsin; max. missed cleavage sites: 2; static modifications: TMTpro (K and peptide N‐terminus) and carbamidomethyl (C); dynamic modifications: oxidation (M), deamidation (N, Q), acetyl (protein N‐terminus), Met‐loss (M) and Met‐loss+Acetyl (M); precursor mass tolerance: 10 ppm; fragment mass tolerance: 0.02 Da.

For reporter ion quantification the most intense m/z in a 20 ppm window around the theoretical m/z was used. Correction of isotopic impurities for reporter ion intensities was applied. Only unique peptides were used for quantification, which was based on S/N values with an average S/N threshold of 10. Normalization was based on total peptide amount and scaling mode on all average.

Only peptides and proteins with FDR < 0.01 were retained while single peptide IDs were excluded from the dataset.

Testing for differentially regulated proteins was performed using R v4.2.0 and the R‐package ‘limma’, using a design without intercept (formula: 0 ~ treatment‐group) and comparisons of interest were extracted using a contrast matrix [38]. The *p*‐values were corrected for multiple hypothesis testing using the method by Benjamini and Hochberg (BH). Only proteins with 3 abundance values per treatment‐group were used for analysis.

In the evaluation of results, we only included proteins of the secretory pathway predicted by SecretomeP1.0, SignalP6.0, and TMHMM2.0 (available at services.healthtech.dtu.dk/services) that were significantly (*p*‐value < 0.05) more or less abundant (Log2 fold change > 1 and < −1) [39, 40, 41]. Pathways were identified using Reactome (available at reactome.org).

## 3 Results

### 3.1 MHC‐I Function in Cell Lines Expressing Variants of Calreticulin and MHC‐I

We aimed to analyse the interaction of calreticulin or oncoCALR and MHC‐I, and assess how a disruption would influence the functionality of MHC‐I. Therefore, we employed co‐culture assays of target cells and effector T cells and used the T cell response as a readout for MHC‐I functionality.

As a target, we used the murine pro‐B cell line Ba/F3 that grows in an IL‐3‐dependent manner before oncogenic transformation and thus represents an ideal model to study growth factor‐independent proliferation after oncogenesis. Furthermore, Ba/F3 cells are derived from the H‐2Kb‐negative C3H mouse strain, as experimentally confirmed in Figure 1A, and thus allow genetic engineering with H‐2Kb variants. Ba/F3 cell lines carrying calreticulin mutations that had previously been generated in our lab were engineered to express H‐2Kb, and mCherry fused to an ovalbumin (OVA)‐derived peptide that is presented by H‐2Kb (Figure 1B,C) [25, 42]. We transduced Ba/F3 cells with retroviral constructs carrying one of three affinity variants of the OVA peptide (high SIINFEKL, medium SIIQFEKL, low SIIVFEKL) and glycosylation site mutants of H‐2Kb (N86Q and N176Q) [43, 44, 45]. This was to test how peptide binding affinity might influence the interaction between calreticulin and MHC‐I, or whether this interaction is solely based on glycosylation and how the expected loss of interaction despite the abundance of CALR might affect H‐2Kb functionality. It is of note that transduction was not controlled for multiplicity of infection (MOI), and potentially varying expression levels of the OVA‐derived peptide might have an influence on MHC‐I‐mediated surface expression [16, 17].

**FIGURE 1:**
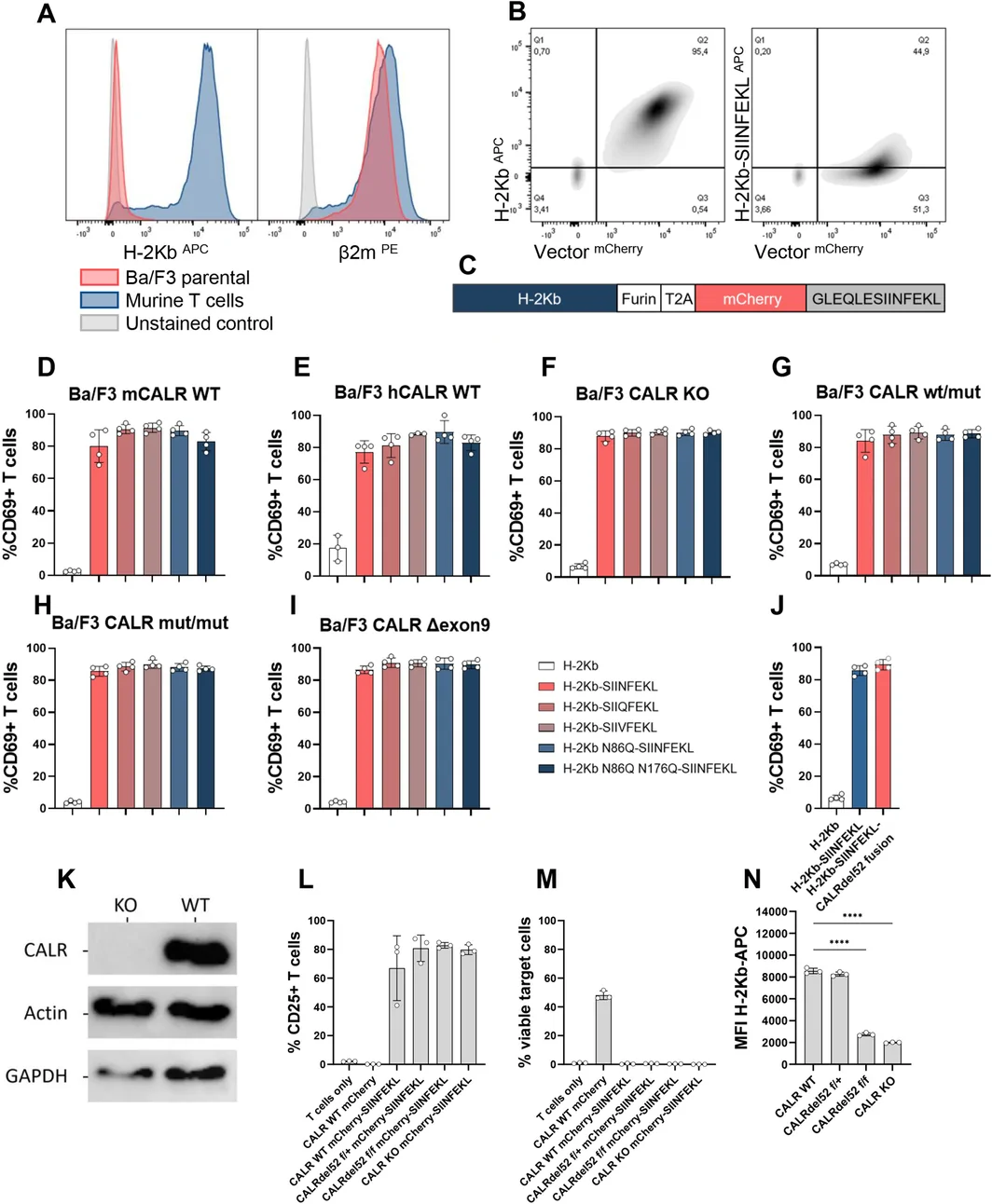
Intact MHC‐I functionality in the Ba/F3 cell line and primary murine haematopoietic cells carrying oncogenic calreticulin mutants or a calreticulin knock out. A–C Engineering of Ba/F3 target cells. (A) Intracellular staining of H‐2Kb and β2m on Ba/F3 cells and T cells as a control. (B) Surface expression of H‐2Kb and presentation of SIINFEKL by Ba/F3 cells after retroviral transduction with H‐2Kb‐mCherry‐SIINFEKL‐pMSCV. (C) Vector map of H‐2Kb‐mCherry‐SIINFEKL‐pMSCV. (D–J) Assessment of T cell activation (%CD69) after co‐culture for 24 h with an effector to target ratio of 1:1 of OT‐I T cells with engineered Ba/F3 target cell lines. Colour annotation for all graphs in I. (D) Target: Expression of wild‐type murine calreticulin. (E) Target: Expression of wild‐type human calreticulin. (F) Target: Calr‐deficient, CRISPR‐Cas9 edited. (G) Target: CRISPR/Cas9 edited oncogenic heterozygous Calr‐del79. (H) Target: CRISPR/Cas9 edited oncogenic homozygous Calr‐del37. (I) Target: Expression of exon 9‐deleted CALR in Ba/F3 cells. (J) Target (red): Ba/F3 Calr‐deficient, transduced to express H‐2Kb, and SIINFEKL that was N‐terminally fused to CALRdel52. (K–N) Assessment of T cell activation (%CD25) after co‐culture for 72 h of OT‐I T cells with engineered primary HSCs. HSCs from different genetic backgrounds were lentivirally transduced to express mCherry‐SIINFEKL. (K) Western blot of whole cell lysates from murine PBMCs obtained from CD57BL/6 N mice that carry wild‐type calreticulin or a calreticulin knock out in the haematopoietic system. (L) OT‐I T cell activation (%CD25) after co‐culture with engineered HSCs. (M) Target cell (HSC) killing after co‐culture with OT‐I T cells. (N) Surface expression of H‐2Kb by murine HSCs.

Engineered Ba/F3 cells were used as a target for OT‐I T cells, which recognize the OVA peptide in the context of H‐2Kb [31].

We co‐cultured engineered Ba/F3 cells and OT‐I T cells and assessed expression of the T cell activation marker CD69 by flow cytometry after 6 and 24 h. We compared the T cell response of OT‐I T cells to Ba/F3 cells carrying wild‐type murine or human calreticulin (Figure 1D,E, Figure S1E,F) with the response to calreticulin mutants. We did not find any defects at any time point in T cell activation after co‐culture with engineered Ba/F3 cells carrying the calreticulin knockout, heterozygous oncogenic mutation, homozygous oncogenic mutation, deletion of exon 9 (Figure 1F–I, Figure S1G–J), overexpression of oncoCALR or Ba/F3 cell lines carrying mutations in the glycan‐binding pocket of calreticulin (Figure S1A–D). Hence, neither oncogenic calreticulin mutations, calreticulin deficiency, antigen affinity nor absence of H‐2Kb glycosylation compromised MHC‐I‐mediated T cell responses and thus highlighted the independence of MHC class I functionality from calreticulin.

### 3.2 Oncogenic Calreticulin Does Not Escape the Peptide Loading Machinery

A previous report claimed the absence of peptides derived from the mutated C‐terminus of oncoCALR from the immunopeptidome, despite those peptides being predicted to be presentable by various common human leukocyte antigen (HLA) alleles, implying an unknown mechanism by which oncoCALR escapes the PLC [21, 22]. We fused the OVA‐derived peptide SIINFEKL to the C‐terminus of oncoCALR and transduced calreticulin‐deficient Ba/F3 cells [42]. Co‐culture with OT‐I T cells resulted in robust CD69 expression (Figure 1J). This suggests that the peptide loading machinery degrades oncoCALR properly and oncoCALR does not escape.

### 3.3 MHC‐I Function in Primary Murine Haematopoietic Cells

Next, we assessed the MHC‐I functionality in primary murine cells. We generated a mouse strain on the C57BL/6N background that is deficient in calreticulin in the haematopoietic system (Figure 1K). Therefore, we crossed mice from a strain carrying a conditional calreticulin knockout with vavCre‐expressing mice [29, 30].

Primary murine haematopoietic stem cells (HSC) were harvested from the bone marrow of hetero‐ and homozygous oncoCALR mice, calreticulin KO mice, and C57BL/6N mice and lentivirally transduced to present the OVA‐derived peptide SIINFEKL by their endogenous H‐2Kb complex [27]. Engineered HSCs were co‐cultured with OT‐I T cells, and expression of the T cell activation marker CD25 and target cell killing were assessed after 72 h. We could not find differences in the T cell activation, measured as the percentage of CD8+ CD25+ T cells, and target engineered HSCs were eradicated in all test groups (Figure 1L,M). We could confirm previous reports of lower surface expression of H‐2Kb by homozygously oncoCALR‐expressing cells and calreticulin‐deficient cells (Figure 1N); however, this does not influence MHC‐I function [14, 15].

### 3.4 Calreticulin Deficiency or Expression of oncoCALR In Vivo Does Not Impair the Antiviral T Cell Response

We wanted to investigate whether our findings from cell lines and primary cells translate to an In Vivo immune response. We infected C57BL/6N mice carrying heterozygous and homozygous oncoCALR, calreticulin knockout, or wild‐type calreticulin in the haematopoietic system under a vavCre promoter or in the liver under an AlbCre promoter with acute LCMV Armstrong [27]. LCMV is an arenavirus that infects most tissues of the host; hence we included both blood‐ and liver‐specific oncoCALR expression to obtain insight into MHC‐I function in different organ systems [46]. Notably, in vavCre mice, also the T cells carry oncoCALR or calreticulin knockout, of which the latter was reported to lead to an activation defect in the calcium response [47]. T cell responses in the blood and spleen were quantified 8 days post‐infection at the peak of the adaptive immune response and when the virus is cleared from the blood of infected WT mice [46]. In the blood and spleen of mice carrying *Calr* mutations in the haematopoietic system, the relative abundance of effector T cells and LCMV‐specific effector T cells was not significantly different (Figure 2A–F). Cytokine production (IFNγ and TNF) of CD8+ T cells harvested from the spleen and stimulated with LCMV peptide gp33 was not altered in *Calr* KO cells but was significantly increased in heterozygous oncoCALR cells (Figure 2G,H), while the cytotoxic protein granzyme B was unaltered in all genotypes (Figure 2I).

**FIGURE 2:**
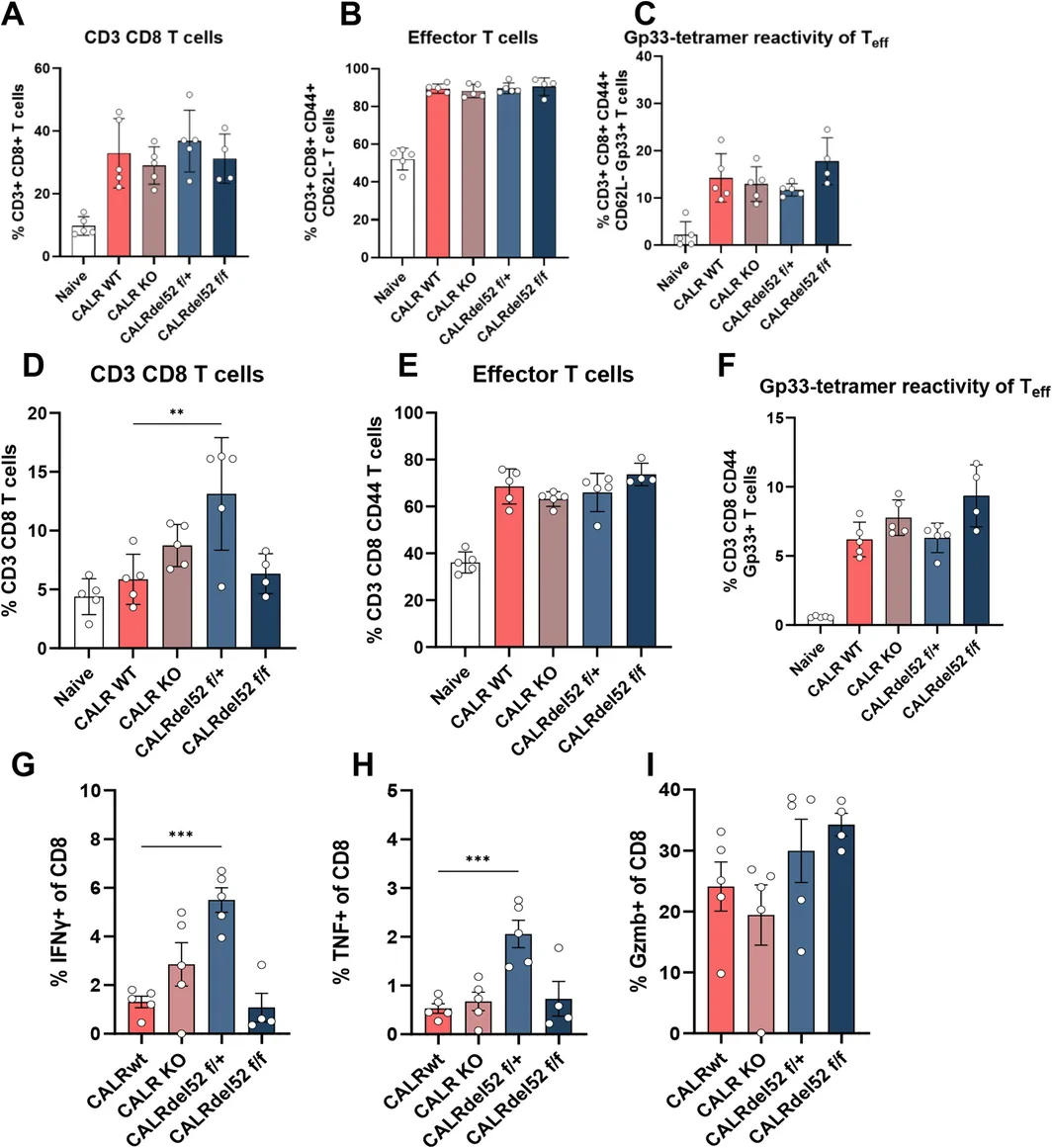
In Vivo T cell response upon LCMV infection in oncoCALR and *Calr* KO mice. Mice carrying calreticulin mutations in the haematopoietic system (controlled by a vavCre promoter) were infected with 2 × 10^6^ focus forming units (ffu) LCMV Armstrong. Cellular and humoral T cell responses were assessed 8 days post infection (dpi) by flow cytometry. (A–C) T cell populations in the blood. (A) Cytotoxic T cell. (B) Effector T cells. (C) Gp33‐reactive effector T cells. (D–F) T cell populations in the spleen. (D) Cytotoxic T cells. E Effector T cells. F Gp33‐reactive effector T cells. (G, H) Intracellular cytokines after re‐stimulation with gp33 in splenic CD8+ T cells. (G) Interferon gamma. (H) Tumour necrosis factor. (I) Granzyme B.

We obtained similar results from mice carrying the *Calr* mutation in the liver (Figure S2) and thus concluded that the absence of calreticulin or the presence of oncogenic calreticulin does not affect the T cell response to a viral pathogen in the alleles that we investigated. Contrary to previous reports, we could not find defective T cell responses in oncoCALR‐carrying and calreticulin‐deficient mice, albeit employing a different marker [47].

### 3.5 MHC‐I Function in Patient‐Derived CALR‐Mutated CD34+ Cells

In the following experiments, we wanted to evaluate MHC‐I function in oncoCALR‐expressing MPN patient samples using a co‐culture assay.

We engineered the Jurkat E6.1 T lymphocyte leukaemia cell line to express a chimeric antigen receptor (CAR) that recognises the endogenous WT1‐derived HLA‐A*02:01‐restricted peptide RMFPNAPYL (Figure 3A,B) [48]. The WT1‐derived peptide was selected as an antigen because WT1 is equally expressed by healthy donor and oncoCALR‐positive MPN patient‐derived CD34+ stem cells that we used as a target in co‐culture experiments [49]. We validated the specificity of the CAR in a co‐culture with the HLA‐A*02:01‐positive megakaryoblastic leukaemia UT‐7 cell line that was engineered to express oncoCALR in the heterozygous or homozygous state, and compared it to UT‐7 cell lines lacking B2M and thus MHC‐I surface expression (Figure 3C). We did not find significant differences between the wild‐type and mutant groups.

**FIGURE 3:**
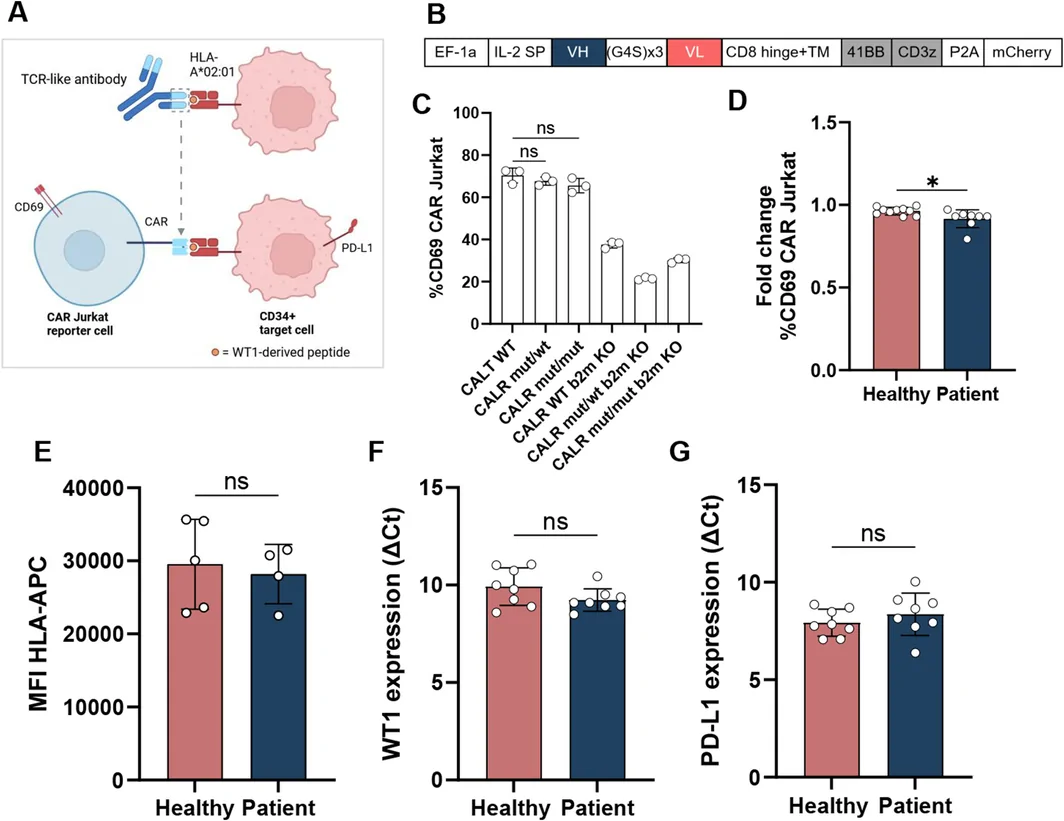
Co‐culture of patient CD34+ cells with a CAR Jurkat reporter cell line. (A) Experimental overview. Jurkats were engineered to express an anti‐HLA‐A*02:01‐WT1 peptide‐BBz CAR. WT1 is endogenously expressed by target cells, and a WT1‐derived peptide is presented by HLA‐A*02:01. (B) anti‐HLA‐A*02:01‐WT1 peptide‐BBz CAR‐pCDH vector design for lentiviral transduction of Jurkat cells. (C) The HLA‐A*02:01‐positive human acute megakaryoblastic leukaemia cell line UT‐7 was CRISPR Cas9‐engineered to express oncoCALR hetero‐ or homozygously. As a control, we engineered HLA‐negative UT‐7 variants by b2m knock out. Engineered UT‐7 cells were co‐cultured with the reporter Jurkat cell line expressing an anti‐HLA‐A*02:01‐WT1 peptide‐BBz CAR. CD69 expression of CAR Jurkat reporter cells after co‐culture with UT‐7 cell lines for 48 h. (D) Target CD34+ cells were isolated from ten HLA‐A*02:01‐ and oncoCALR‐positive patients, and eight HLA‐A*02:01‐positive healthy control donors. The graph shows Jurkat activation (CD69 expression) after 48 h of co‐culture of CAR Jurkats and patient‐ or healthy control‐derived CD34+ cells. In this graph, data is presented as a fold change (normalized to the highest CD69‐expressing healthy control donor) of CD69 surface expression, thereby combining the results from two experiments due to low patient sample abundance. (E) HLA surface expression by CD34+ cells. (F) WT1 expression levels in target CD34+ cells. (G) PD‐L1 expression levels in target CD34+ cells.

CD34+ stem cells were isolated from HLA‐A*02:01‐ and oncoCALR‐positive patients and healthy control donors. Patient samples were characterized by RNA sequencing in a previous study and variant allele frequency (VAF) had been determined at a median of 0.34 (min. 0.22, max. 0.43) at the time of sample acquisition [21]. CAR Jurkat T cells and expanded CD34+ cells were co‐cultured and expression of the T cell activation marker CD69 was assessed after 48 h. We found a marginally lower CD69 expression in CAR Jurkat T cells co‐cultured with patient‐derived target cells (Figure 3D, Figure S3A). This significant difference in the primary cells, however, was not reflected by the results from the cell line (Figure 3C). Nevertheless, we tried to detect a cause for this decrease but could not find differences in HLA surface expression, antigen expression, or expression of the T cell‐suppressing ligand PD‐L1 by target CD34+ cells from patients and controls (Figure 3E–G). We therefore concluded that MHC‐I function is intact in oncoCALR‐expressing human cell lines and minimally, if at all, impaired in MPN patient‐derived HLA‐A*02:01‐expressing CD34+ cells.

### 3.6 Calreticulin Is an Indispensable Chaperone for a Subset of Proteins but Not MHC‐I

After all preliminary results, surprisingly, hinted at the negligible influence of calreticulin on MHC class I functionality, we aimed to pinpoint its actual role as a chaperone at the proteome level. Therefore, we performed tandem mass tag mass spectrometry (TMT‐MS) to identify proteins that were missing or significantly decreased in abundance. We analysed whole‐cell lysates from primary murine neutrophil granulocytes and cytotoxic T cells obtained from *Calr* knockout and homozygous oncoCALR mice and compared them with samples from wild‐type C57BL/6N mice. Neutrophils were selected because previous studies in neutrophils from human homozygous oncoCALR patients found myeloperoxidase (Mpo) to be missing [50, 51]. We wanted to further investigate whether this absence also holds true for calreticulin‐deficient cells and whether there is an overlap of proteins missing between the loss‐of‐function calreticulin deficiency and the gain‐of‐function oncogenic calreticulin mutation. T cells were included because of reported activation defects in calreticulin‐deficient murine lymphocytes that we wanted to investigate on the proteomic level [47].

In the evaluation of results, we only included proteins that were predicted to be part of the secretory pathway because only these proteins can potentially interact with the ER‐based chaperone calreticulin.

We identified five absent proteins in samples from both test genotypes and cell types, indicating that calreticulin is their essential chaperone: acyloxyacyl hydrolase (Aoah), eosinophil peroxidase (Epx), neutral alpha‐glucosidase AB (Ganab), myeloperoxidase (Mpo) and phospholipase B‐like 1 (Plbd1) (Figure 4A–E, Figure S4A–D). The abundance of murine MHC‐I (H‐2 Kb) remained steady (Figure 4A,B, Figure S4A,B). These missing proteins belong to pathways related to immunity and metabolism, as identified by Reactome (Figure 4F).

**FIGURE 4:**
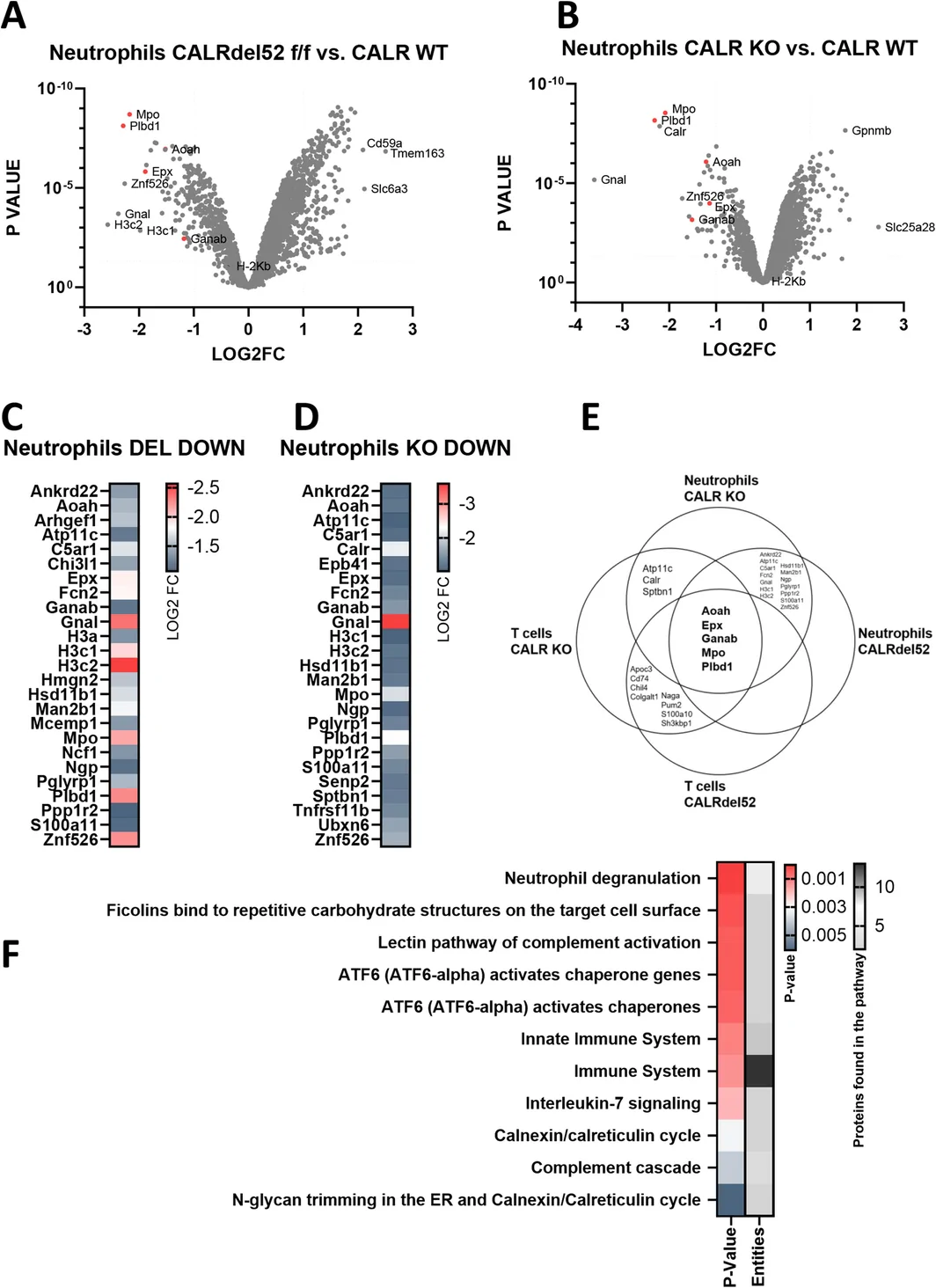
Role of calreticulin in the proteome of murine neutrophil granulocytes. (A) differentially abundant protein of the secretory pathway in homozygous oncoCALR‐carrying neutrophils compared to wild‐type. Proteins that are missing in all an analysed cell types and genotypes are highlighted in red. (B) Differentially abundant proteins of the secretory pathway in calreticulin deficient cells compared to wild‐type. Proteins that are missing in all an analysed cell types and genotypes are highlighted in red. (C) Top 25 less abundant proteins in homozygous oncoCALR‐carrying neutrophils among significantly differently (*p*‐value < 0.05) expressed proteins. (D) Top 25 less abundant proteins in calreticulin deficient neutrophils among significantly differently (*p*‐value < 0.05) expressed proteins. (E) Overlaps of missing proteins, neutrophils and CD8+ T cells and oncoCALR and CALR‐deficiency. (F) Affected pathways in calreticulin‐deficient neutrophils.

We also found proteins missing in a cell type‐specific fashion, for example neutrophilic granule protein (Ngp) in neutrophils (Figure 4C–E) or the MHC class II‐associated invariant chain (Cd74) in T cells (Figure 4E, Figure S4A–D).

Despite decreased surface expression (Figure 1N) we could not find significant changes in the abundance of H‐2Kb in oncoCALR‐expressing or CALR KO cells in the proteome, which is likely because H‐2Kb accumulates in the Golgi apparatus [18].

We concluded that calreticulin is an integral chaperone for a few specific proteins, but not MHC‐I.

## 4 Discussion

In this study, we originally aimed to elucidate the influence of oncogenic calreticulin variants on MHC‐I functionality but instead found that calreticulin is expendable for MHC‐I functionality altogether.

We implemented various experimental models that employed T cell responses to known antigens as the main indicator of intact MHC‐I function. Using murine cell lines and primary cells, we assessed the response of OT‐I T cells to the OVA‐derived peptide and its affinity variants and could not find any difference between mutated and wild‐type calreticulin‐expressing cells. Furthermore, removal of N‐glycosylation sites (N86 and N176) from H‐2Kb did not affect antigen recognition by the T cells, confirming a previous study by Barbosa [43]. Supporting the in vitro data, evaluation of the T cell compartment of mice after infection with LCMV did not reveal significant differences between groups. Patient‐derived CD34+ cells co‐cultured with a CAR reporter cell line could elicit only a slightly decreased activation level compared with healthy control donors, albeit no significant difference could be found in a cell line. We used cells and mice deficient in calreticulin as controls. Surprisingly, we observed no impairment of MHC‐I functionality in calreticulin‐deficient cells.

We consider the discovery that MHC‐I functionality is independent of calreticulin and its oncogenic variants the principal finding of this study because calreticulin had been considered a crucial component of the PLC of MHC‐I for decades [3, 4, 5, 6, 7].

How was calreticulin's role in MHC‐I function misinterpreted? The association of calreticulin and MHC‐I came from co‐immunoprecipitation assays from human cell lines conducted by Sadasivan et al. in 1996 and later confirmed by Rufer and Leonhardt [6, 7]. Blees et al. identified calreticulin as a part of the PLC by cryo‐electron microscopy from affinity‐purified extracts from human cell lines [3]. We assume that calreticulin was found associated with MHC‐I in these studies because it is a lectin chaperone that can interact with any glycoprotein spawning a Glc1Man9GlcNAc2 oligosaccharide and thus contaminates affinity purification‐based analysis [52]. Not surprisingly, calreticulin is a member of the ‘CRAPome’, a repository listing nonspecific binding partners that are frequently found in co‐immunoprecipitation/LC–MS protein interaction studies [53]. Calreticulin may maintain an interaction with MHC‐I, but this does not influence its function. Moreover, Zernich et al. even reported HLA alleles that are completely independent of the entire peptide loading complex [54].

Nevertheless, calreticulin does play an important role in embryogenesis and reproduction, as pan‐calreticulin‐deficient mice are not viable due to a defect in cardiogenesis, and female mice that produce calreticulin‐deficient oocytes are infertile [30, 55]. We also observed in our animal husbandry that female mice that express oncoCALR homozygously or a *Calr* KO in their haematopoietic system under the control of a vavCre promoter do not breed.

After failing to prove a pivotal role of calreticulin in MHC‐I functionality, we wanted to elucidate whether the chaperone calreticulin is an essential factor for other proteins. Therefore, we performed TMT‐MS on primary murine neutrophils and T cells that carried a homozygous oncogenic calreticulin mutation or were deficient in calreticulin and identified differences in the proteome compared with wild‐type. We identified an overlap of proteins missing in all groups, among which were acyloxyacyl hydrolase (Aoah), eosinophil peroxidase (Epx), neutral alpha‐glucosidase AB (Ganab), myeloperoxidase (Mpo), and phospholipase B‐like 1 (Plbd1), suggesting calreticulin as a crucial chaperone in their formation. Mpo was already identified previously to be absent in patients with a rare homozygosity for the common oncogenic calreticulin mutation [50, 51]. Several of the missing proteins are involved in the antibacterial immune response, thereby implying an immune defect in patients; however, studies on such immune deficiency have yet to be conducted.

Furthermore, our findings encourage the feasibility of cancer vaccination for MPN patients. Previous studies failed to develop such a vaccine, claiming defects in the MHC‐I functionality as a cause [56, 57].

In conclusion, our study provides evidence that the importance of calreticulin for proper MHC‐I functionality, at least in the alleles that we investigated, had been massively overestimated. Our results suggest that calreticulin is not essential for the functional capacity of MHC‐I to present peptide antigens, although it does influence the abundance of MHC‐I molecules at the cell surface. A potential mechanism for the lower surface expression could be retention of MHC‐I molecules in the Golgi apparatus after loss of the endoplasmic retention signal caused by the altered amino acid sequence in oncoCALR or absence of calreticulin altogether [18]. Adding to the perception that calreticulin has a quantitative rather than qualitative influence on MHC‐I functionality, it was also reported to influence levels of surface‐presented peptides [16, 17].

Calreticulin deficiency decreases the surface levels, but reports show that even a single MHC‐I molecule triggers a T cell response via the MHC/TCR interaction [58].

Thus, the functional role of calreticulin in the peptide loading complex needs to be redefined and its significance reevaluated.

## Author Contributions

**Maria Höhrhan:** investigation, writing – original draft, visualization, formal analysis, project administration, data curation, writing – review and editing, validation. **Laura Antonio‐Herrera:** investigation, writing – review and editing, formal analysis. **Henrique G. Colaço:** investigation, writing – review and editing, formal analysis. **Anja Wagner:** investigation, writing – review and editing. **Markus Unterwurzacher:** investigation, software, formal analysis, visualization. **Cecilia Varga:** investigation. **Klaus Kratochwill:** methodology, writing – review and editing, supervision, resources. **Andreas Bergthaler:** methodology, writing – review and editing, supervision, resources, funding acquisition. **Robert Kralovics:** methodology, writing – review and editing, supervision, resources, funding acquisition, conceptualization, project administration, validation.

## Funding

Funding for this study was provided by Austrian Science Fund grants number P34451‐B, P35806‐B, and P36646‐B. HGC received funding from the European Union's Horizon 2020 research and innovation program under Marie Skłodowska‐Curie Actions, grant agreement No 10102897.

## Ethics Statement

MPN patient and healthy control donor samples were collected with informed consent under the approval #553/2008 of the ethics committee of the Medical University of Vienna.

In Vivo experiments were approved by the Austrian Federal Ministry of Education, Science and Research in animal experimentation licences 2020–0.448.862 and 2020–0.406.011.

## Conflicts of Interest

Robert Kralovics is a co‐founder of MyeloPro Diagnostics and Research GmbH. Klaus Kratochwill is a consultant of Zytoprotec GmbH. All other authors declare no conflicts of interest.

## Supporting information


**Figure S1:** Co‐culture of OT‐I T cells and Ba/F3 cell line.
A–D. Assessment of T cell activation (% CD69+) after co‐culture for 24 h of OT‐I T cells with engineered Ba/F3 cell lines. Colour‐code annotation for graphs in panels A–D is shown in panel J.
A. Target cells: Overexpression (OE) of oncogenic hCALRdel52 in Ba/F3.
B–D. Target cells: Overexpression of oncogenic hCALRdel52 with different mutations in the glycan‐binding domain of Calr.
E–J. Flow cytometric assessment of T cell activation (% CD69) after 6 h of co‐culture OT‐I T cells with engineered Ba/F3 cell lines. Colour‐code annotation for graphs in panels E–J in panel J.
E. Target: Expression of wild‐type murine calreticulin.
F. Target: Expression of wild‐type human calreticulin.
G. Target: Calr‐deficient, CRISPR‐Cas9 edited.
H. Target: CRISPR/Cas9 edited oncogenic heterozygous Calr‐del79.
I. Target: CRISPR/Cas9 edited oncogenic homozygous Calr‐del37.
J. Target: Expression of exon 9‐deleted CALR in Ba/F3 cells.
K. Comparison of the T cell activation (% CD69+) of CD8+ OT‐I T cells after 24 h of co‐culture with Ba/F3 cells transduced with H‐2Kb and either only the SIINFEKL peptide or full length ovalbumin (OVA).
L. Gating strategy used for analysis of flow cytometry results from co‐culture experiments using the Ba/F3 cell line or Lin‐ primary murine cells as target cells, and OT‐I T cells as effector cells.
**Figure S2:** In vivo T cell response upon LCMV infection in oncoCALR and CALR KO mice
Mice carrying Calr mutations in the liver (controlled by an AlbCre promoter) were infected with 2 × 10^6^ focus forming units (ffu) of LCMV Armstrong. Cellular and secretory T cell responses were assessed 8 days post‐infection (dpi) by flow cytometry.
A–C. T cell populations in the blood.
A. Cytotoxic T cells.
B. Effector T cells.
C. Gp33‐reactive effector T cells.
D–F. T cell populations in the spleen.
D. Cytotoxic T cells.
E. Effector T cells.
F. Gp33‐reactive effector T cells.
G, H. Intracellular cytokines after re‐stimulation with gp33 in cytotoxic T cells in the spleen.
G. Interferon gamma.
H. Granzyme B.
I. Tumour necrosis factor.
J. Mice body weight at the start of and during LCMV infection.
**Figure S3:** Co‐culture of patient CD34+ cells with a CAR Jurkat reporter cell line.
A. Individual graphs of two experiments that are depicted in Figure 3D normalized to the highest measured value in each experiment.
**Figure S4:** Role of calreticulin in the proteome of murine CD8+ T cells.
A. Differentially abundant proteins of the secretory pathway in homozygous oncoCALR‐carrying CD8+ T cells compared to wild‐type. Proteins that are missing in all an analysed cell types and genotypes are highlighted in red.
B. Differentially abundant proteins of the secretory pathway in calreticulin‐deficient CD8+ T cells compared to wild‐type. Proteins that are missing in all an analysed cell types and genotypes are highlighted in red.
C. Top 25 less abundant proteins in homozygous oncoCALR‐carrying CD8+ T cells among significantly differentially (*p*‐value < 0.05) expressed proteins.
D. Top 25 less abundant proteins in calreticulin deficient CD8+ T cells among significantly differentially (*p*‐value < 0.05) expressed proteins.
E. Affected pathways in calreticulin‐deficient CD8+ T cells.

## Data Availability

All unique/stable reagents (e.g., plasmids) generated in this study are available from the corresponding author with a materials transfer agreement. All data reported in this paper will be shared by the corresponding author upon request. The data that support the findings of this study are openly available in PRIDE at https://www.ebi.ac.uk/pride/, reference number PXD062085.
